# High sensitivity infrared gas sensing and filtering using metasurfaces plasmonic ring resonators

**DOI:** 10.1371/journal.pone.0353973

**Published:** 2026-10-27

**Authors:** El-Aouni Mimoun, Ali Hennache, Mahmoud A. Zaky, Ben-Ali Youssef, Essahlaoui Abdelouahed, Bria Driss, Ali Alzahrani, Zaky A. Zaky

**Affiliations:** 1 Laboratory of Materials, Waves, Energy and Environment, Team of Waves, Acoustics, Photonics and Materials, Mohamed I University, Oujda, Morocco; 2 Deanship of Scientific Research, Imam Mohammad Ibn Saud Islamic University (IMSIU), Riyadh, Saudi Arabia; 3 Department of Mathematics and Statistics, College of Science, Imam Mohammad Ibn Saud Islamic University (IMSIU), Riyadh, Saudi Arabia; 4 Engineering Sciences Laboratory (LSI), Multidisciplinary Faculty of Taza, Sidi Mohamed Ben Abdellah University, Taza Gare, Morocco; 5 Department of Physics, Al-Qunfudah University College, Umm Al-Qura University, Makkah, Saudi Arabia; 6 Physics Department, Faculty of Science, Beni-Suef University, Beni Suef, Egypt; 7 Department of Mathematics and Natural Sciences, Gulf University for Science and Technology, Mishref, Kuwait; National Research Centre, EGYPT

## Abstract

This work investigates a plasmonic metal–insulator–metal (MIM) ring structure periodically coupled to dual resonators and a defect cell, engineered for infrared filtering and gas sensing. The optical response is modeled numerically using the finite element method in COMSOL Multiphysics, enabling accurate analysis of strong field confinement and loss in the subwavelength MIM geometry. By tuning the geometrical parameters of the rectangular and lateral resonators and of the defect cavity, we show that the transmission spectrum, band-gap position, and defect resonance wavelength can be precisely controlled, and that introducing a defect resonator into a periodic dual-resonator array generates a sharp, highly localized mode inside the plasmonic band gap. Small variations of the refractive index in the gas-filled device lead to clear, nearly linear shifts of this defect resonance, yielding a high sensitivity of about 1920 nm/RIU together with a Q-factor above 100 and a low detection limit, so that sensitivity, spectral selectivity, and detection capability are simultaneously enhanced. These results highlight the novelty of combining periodic band-gap engineering, dual-resonator coupling, and defect optimization in a single MIM platform, and demonstrate that the proposed structure can function as both a narrowband photonic filter and a high-performance infrared gas sensor in a compact, integrated configuration.

## 1. Introduction

Advances in photonics and the manipulation of optical materials have enabled new prospects for the development of new applications such as filters, demultiplexers and sensors based on resonant structures [1–8]. Such devices are important components for many applications, such as communication, detection and signal processing [9–17]. One class of structures used to fabricate these devices, rings and resonators, has gained increasing interest. These components can filter specific frequencies, exhibit high-quality factors, and be customized to order. By exploiting geometric parameters, such as the size and shape of the structures, and optical parameters, such as the refractive index of the materials used, their performance can be finely tuned, providing flexible and efficient solutions for a variety of applications [18–22]. The appropriate choice of these parameters is important for optimizing the properties of the structures, whether in terms of transmission, frequency selectivity, quality factor (Q-factor), or sensitivity in detection applications [23–26].

In addition, the use of platforms such as silicon-on-insulator, polymers and plasmonic materials in photonic devices based on ring resonator structures in particular, these devices offer high sensitivity and easy integration into photonic systems. So, these structures have applications in biomedical sensing, environmental monitoring and chemical analysis, although their fabrication remains challenging, particularly at the nanoscale [27–29]. Similarly, compact sensors based on closed-loop resonators are of particular interest in specific applications, such as the detection of food adulterations, thus meeting growing quality control requirements. These technological developments in photonics, combining theory and practical applications, demonstrate the enormous potential of this discipline to transform various sectors and meet today’s global challenges [30,31]. A promising contribution of these advancements is the creation of ultra-fine filters that leverage surface polaritons to achieve enhanced sensitivity. These devices are particularly useful in surface resonance sensors, for detecting biological and chemical samples with remarkable precision [32].

Besides, optical nanofibers generated by adiabatic stretching of conventional fibers enable high localization of optical fields and minimize losses. These nanofibers has paved the way for advanced devices, including high-quality resonators and ultra-sensitive sensors that exploit evanescent fields, enabling greater precision and reliability in complex environments [33]. Besides, the light-matter interactions explored in advanced photonic resonators have enabled the emergence of innovative phenomena, such as continuum bound states, which result from destructive interference between resonant modes in coupled optical cavities. These states can be converted into useful resonances by perturbation, giving rise to effects such as Fano resonance and electromagnetically induced transparency. These advances open up new possibilities for sensors, lasers and other photonic devices [34–39].

Surface plasmon resonance offers the possibilities for light manipulation, particularly for optical filtering and high sensitivity sensing applications. By tuning the properties of plasmonic materials, it becomes possible to design devices capable of selecting, amplifying or blocking specific wavelengths of light, paving the way for advanced technologies in fields such as optical sensors, biosensors and spectral filters [40–43]. In this context, MIM (Metal-Insulator-Metal) waveguides enable light to be guided at the nanoscale through the excitation and confinement of surface plasmons. MIM waveguides are particularly effective at transmitting light at size scales below the wavelength of light, which is essential for the miniaturization of plasmonic devices. Their ability to propagate surface plasmons makes them particularly suitable for plasmonic biosensors and specific substance detection systems, exploiting minimal changes in refractive index [44–51].

Recent studies on MIM waveguide-based plasmonic sensors have demonstrated highly sensitive refractive index sensing using complex resonator geometries and Fano resonances, but most of these designs do not incorporate explicit band-gap and defect-mode engineering [52,53]. In parallel, several defect-engineered one-dimensional photonic crystal sensors and defect-layer devices have been proposed, leveraging photonic band gaps and localized defect states for enhanced sensitivity in biosensing, chemical and environmental monitoring.

In contrast to previously reported photonic crystal filters and MIM plasmonic sensors based on single resonators or simple ring configurations, the present work introduces a periodic MIM waveguide architecture integrating a primary rectangular resonator, a lateral secondary resonator, and an engineered defect cell. This combination enables simultaneous bandgap formation, strong field localization at the defect, and tunable defect modes with high spectral separation, which collectively result in a markedly enhanced sensitivity and detection limit for infrared gas sensing. The comparative data in Table 2 show that the proposed device surpasses earlier designs in both sensitivity and detection limit, underscoring its potential as a high-performance infrared gas sensor and filter.

**Table 1:** Structural parameters.

Parameter	Value (nm)	Parameter	Value (nm)
${\text{w}}_{\text{1}}$	100	${\text{h}}_{\text{1}}$	500
${\text{w}}_{\text{2}}$	50	${\text{h}}_{\text{2}}$	725
${\text{w}}_{\text{3}}$	50	${\text{h}}_{\text{3}}$	625
${\text{w}}_{\text{0}}$	100	${\text{h}}_{\text{4}}$	1500
${\text{d}}_{\text{1}}$	1275	${\text{h}}_{\text{0}}$	500
${\text{d}}_{\text{2}}$	625	${\text{d}}_{\text{0}}$	${\text{d}}_{\text{1}}/\text{2}$

**Table 2:** Comparative analysis of the proposed detector with prior designs.

Year	Structure	DL (RIU)	S (nm/RIU)
2015, [44]	Tooth-shaped plasmonic resonator	0.01	733
2016, [47]	MIM-coupled double rectangular cavities	0.01	596
2016, [63]	Photonic crystal micro-cavity	$\text{1}.\text{5}\times {\text{1}\text{0}}^{-\text{6}}$	500
2018, [50]	MIM with dual side-coupled ring resonators	0.02	1114
2019, [51]	MIM with circular split-ring resonator cavity.	0.03	350
2020, [46]	MIM with cross-shaped cavity	0.02	1075
2023, [64]	Step/graded index photonic crystal resonator	$\text{2}.\text{6}\times {\text{1}\text{0}}^{-\text{3}}$	1000
2023, [65]	Heterostructure-based topological nanophotonic	$\text{2}.\text{2}\times {\text{1}\text{0}}^{-\text{6}}$	774
2024, [66]	Chirped graded photonic crystal	$\text{2}\text{0}\text{3}\times {\text{1}\text{0}}^{-\text{6}}$	410
2024, [49]	MIM plasmonic sensor	–	1638
2024, [67]	Graded-index topological resonator	$\text{2}\text{2}\times {\text{1}\text{0}}^{-\text{6}}$	852
2025, [48]	MIM with square ring resonator	0.01	1600
2026, [68]	MIM with semicircular resonator	$\text{2}.\text{2}\times {\text{1}\text{0}}^{-\text{5}}$	452
2026, [69]	MIM with triangular cavity and elliptical island	$\text{8}.\text{3}\times {\text{1}\text{0}}^{-\text{7}}$	1207
2026, [70]	MIM with rectangular waveguide	–	1113
**2026, This work**	**Periodic MIM with rectangular resonator**	$2.5\times 10^{-4}$	**1920**

## 2. Theoretical model

The unit cell of the proposed 2D plasmonic structure, illustrated in Fig 1a, consists of a metal-insulator-metal (MIM) waveguide coupled with a primary rectangular resonator and a secondary lateral resonator. The main waveguide divides the rectangular resonator into two distinct sections, with the lateral resonator connected to the upper portion. The rectangular resonator, main waveguide, and lateral resonator are filled with an insulating material (initially air, which will later be replaced by different gas samples for sensing applications). These components are entirely encapsulated by silver, which serves as the plasmonic material. Fig 1b depicts the periodic arrangement of the unit cells without any structural defects, while Fig 1c introduces a defect cell, where only the lateral resonator is retained, modifying the transmission properties for enhanced sensing capabilities.

**Fig 1:**
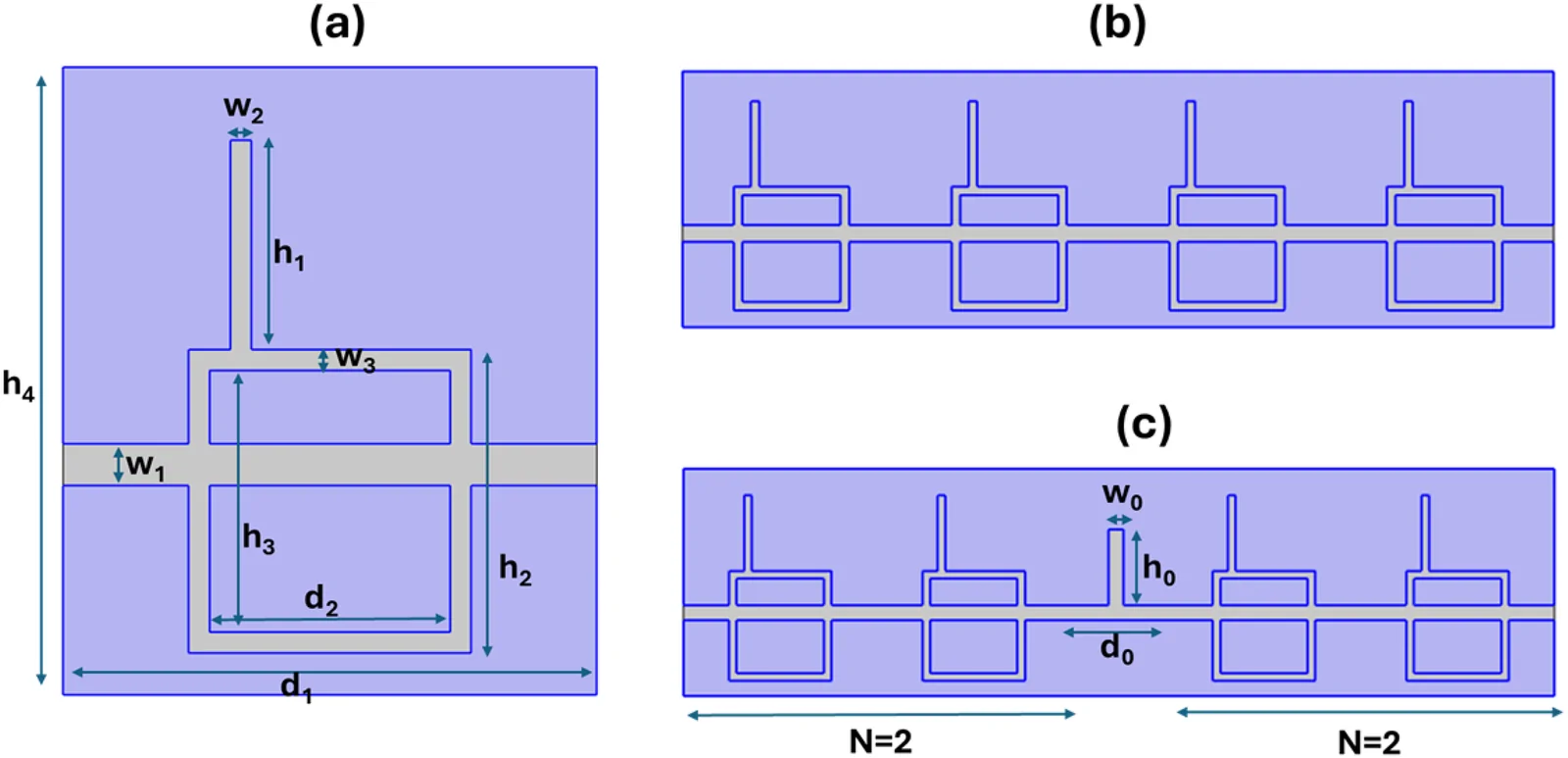
Schematic illustration of (a) the unit cell of the proposed plasmonic sensor, comprising a MIM waveguide coupled with a primary rectangular resonator and a secondary lateral resonator; (b) the periodic structure formed by repeating the unit cell without defects; and (c) the modified periodic structure with a defect cell, where only the lateral resonator is retained.

Unlike conventional MIM plasmonic sensors that typically employ a single ring or cavity side-coupled to a straight waveguide [49], the proposed structure combines a periodic MIM waveguide with a primary rectangular resonator and a lateral secondary resonator, and then introduces a defect cell in the periodic chain. This dual-resonator periodic architecture allows simultaneous band-gap formation and defect mode localization, which cannot be achieved with a single-ring MIM sensor or standard one-dimensional photonic crystal filters. The repetition of the unit cell along the propagation direction leads to the formation of plasmonic band gaps, analogous to photonic band gaps in 1D photonic crystals, but here it is realized in a compact MIM waveguide platform. In contrast to conventional multilayer photonic crystals based on alternating dielectric stacks, the present periodic system relies on coupled plasmonic resonators embedded in a MIM waveguide, which enables subwavelength confinement and easier integration with on-chip plasmonic components. The introduction of a defect cell, where only the lateral resonator is retained, creates a localized state inside the plasmonic band gap. By adjusting both the position of the defect (N) and its geometric parameters (w₀, h₀), it becomes possible to tailor the spectral position and isolation of the defect mode, which is crucial for high-sensitivity refractive index sensing. This type of controlled defect engineering within a periodic MIM–resonator metasurface is distinct from previously reported single-defect photonic crystals and MIM ring sensors.

The optical response of the proposed plasmonic structure, including reflectance (R), transmittance (T), and absorption (A), was numerically simulated using the Finite Element Method (FEM). FEM is chosen for its ability to handle complex geometries and accurately model electromagnetic wave interactions at subwavelength scales, particularly in plasmonic systems where near-field effects dominate. The equations describe the finite element formulation for boundary mode analysis of electromagnetic waves in plasmonic structure, focusing on the electric field (E) propagation and decay, as follows [54]:

For the waveguide and resonators (Air medium):


$$\begin{array}{c} \nabla \times (\nabla \times \text{E})-{\text{k}}^{\text{2}}\varepsilon_{air}E=\text{0} \end{array}$$ (1)

where k =ω/c is the free-space wavenumber, $\varepsilon_{r}$ is the relative permittivity for air or the gas sample, and E is the electric field vector.

For metal (silver):


$$\begin{array}{c} \nabla \times \mu_{m}^{-\text{1}}(\nabla \times \text{E})-{\text{k}}^{\text{2}}\varepsilon_{m}E=\text{0} \end{array}$$ (2)

The electric field is assumed to separate into transverse (x,y) and longitudinal (z) components:


$$\begin{array}{c} \text{E}(x,y,z)=E(x,y){\text{e}}^{-\alpha z} \end{array}$$ (3)

where α (decay constant) and β (propagation constant) are combined as:


$$\begin{array}{c} \alpha =j\beta =-\lambda \end{array}$$ (4)

where λ is the eigenvalue solved by FEM.

The optical response of the structure is computed using the Electromagnetic Waves, Frequency Domain (ewfd) interface in COMSOL, solving the full-vector wave equation in 2D for the electric field. The model employs numeric Port boundaries at the input and output facets of the MIM waveguide to excite and collect the fundamental TM mode, while the remaining external boundaries of the computational window are treated as perfect electric conductors (PEC), so that the fields are confined inside the simulation region. Internal metal–dielectric interfaces use the default continuity conditions, ensuring continuity of the tangential field components.

The complex permittivity of silver is modeled using COMSOL’s Drude–Lorentz dispersion model with only a single Drude term activated. In this configuration the relative permittivity is:


$$\begin{array}{c} \varepsilon_{m}=\varepsilon_{\infty }-\frac{\omega_{p}^{\text{2}}}{\omega^{\text{2}}+j\gamma \omega } \end{array}$$ (5)

Here, $\varepsilon_{\infty }=\text{3}.\text{7}$ represents the permittivity at infinite frequency; ω denotes the angular frequency of incident electromagnetic waves; $\gamma ={\text{2}.\text{634}\times \text{10}}^{\text{13}}\;rad/s$ is the electron collision frequency; and $\omega_{p}={\text{1}.\text{38}\times \text{10}}^{\text{16}}\;rad/s$ rad/s corresponds to the silver’s bulk plasma frequency.

Physically, the resonances of the proposed structure originate from constructive interference of surface plasmon polaritons circulating in the coupled rectangular and lateral resonators and interacting with the MIM waveguide. The resonance condition is governed by the effective optical path length in the cavities, which depends on both their geometrical dimensions and the effective refractive index of the metal–insulator region. Any variation in the refractive index of the gas or in the resonator dimensions therefore changes the accumulated phase along a round trip and shifts the resonance wavelength, which underpins the sensing mechanism [27,55].

When the unit cell is periodically repeated, the interference between forward and backward propagating plasmonic modes gives rise to plasmonic band gaps, in which propagation through the structure is strongly suppressed, similar to photonic band gaps in dielectric photonic crystals. Introducing a defect cell breaks the periodicity and creates a localized plasmonic mode whose frequency falls inside the band gap; this defect mode is spatially confined around the defect and decays into the periodic environment, enabling strong field localization and high sensitivity to local refractive index changes [56].

Ethics Declarations: This article does not contain any studies involving animals or human participants performed by any authors.

## 3. Result and discussions

Section 3 is organized as follows: first we analyze the field distribution and basic resonance behavior in a single unit cell, then we investigate band-gap formation in the periodic structure, followed by the effect of introducing and optimizing a defect cell, and finally we evaluate the sensing figures of merit and compare them with previously reported plasmonic sensors. The FEM is used to study the electric field distribution (EFD), transmittance, reflectance, and absorbance inside the proposed 2D plasmonic structure consists of a periodic MIM waveguide coupled with a primary rectangular resonator and a secondary lateral resonator. The initial conditions are presented in Table 1.

### 3.1 Wave propagation characteristics of a 2D plasmonic unit cell

Fig 2 shows the electric field distribution in one unit cell of the proposed structure at three representative wavelengths: 1624 nm (Fig 2a), 2100 nm (Fig 2b), and 3000 nm (Fig 2c). At λ = 1624 nm, where the transmission coefficient reaches its maximum and reflection is nearly zero (as clear in Fig 3), the field is strongly confined along the MIM waveguide and efficiently coupled into the rectangular and lateral resonators, indicating a well-excited transmission resonance. At λ = 2100 nm, the field pattern is less uniformly distributed: a significant part of the field remains near the input and in the resonator region, consistent with partial transmission, increased reflection and a noticeable absorption peak in the spectra. At λ = 3000 nm, the field is mainly concentrated near the input side and decays rapidly along the structure, in agreement with the high reflection and very low transmission observed in Fig 3, which shows that the wave is predominantly reflected at this wavelength.

**Fig 2:**
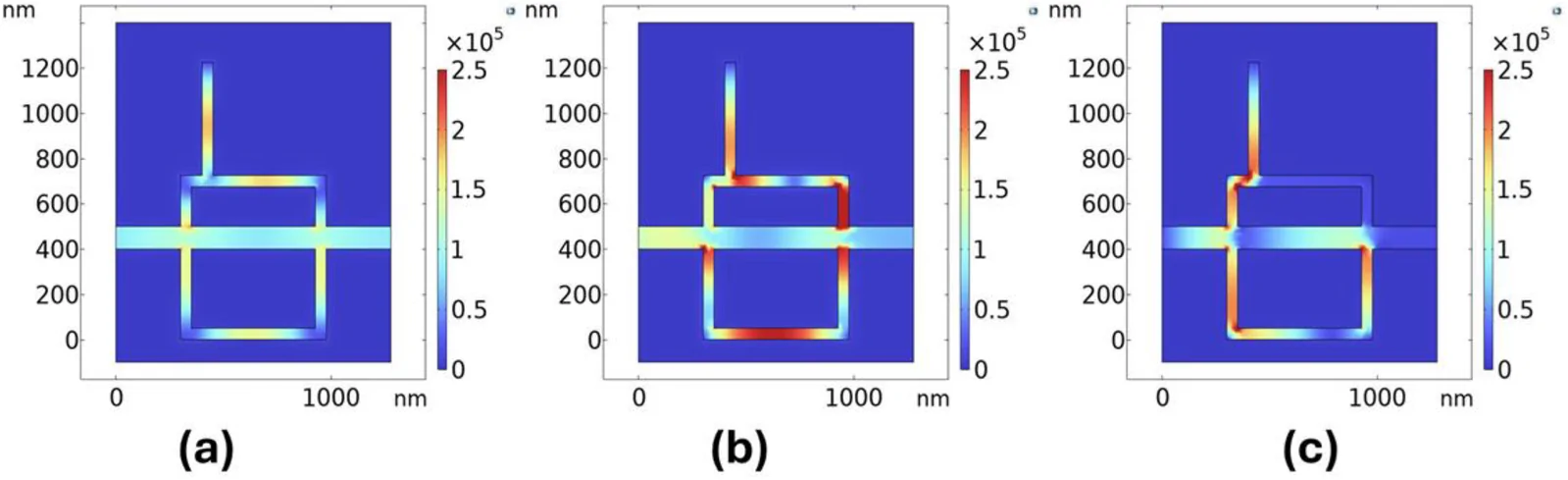
The EFD (V/m) in one unit cell of the proposed structure at (a) 1624 nm, (b) 2100 nm, and (c) 3000 nm. The color scale indicates the local field amplitude in the metal–insulator–metal channel and around the resonator regions for each excitation wavelength.

**Fig 3:**
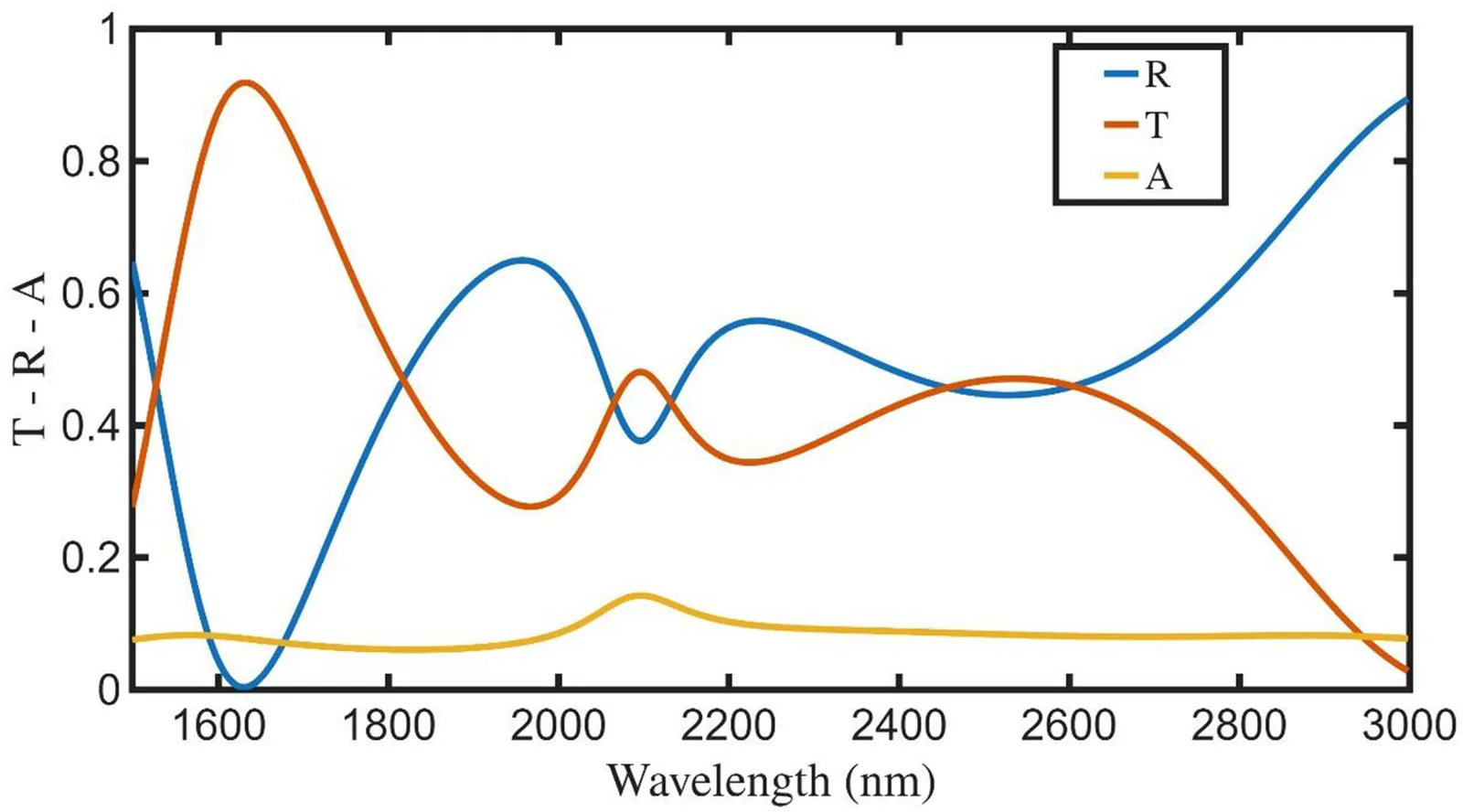
Transmission (T), reflection (R), and absorption (A) spectra of a single unit cell of the proposed plasmonic structure shown in Fig 1a, plotted as a function of wavelength in the range 1500–3000 nm.

Fig 3 illustrates the spectral variations of the transmission (T), reflection (R), and absorption (A) coefficients of the plasmonic structure proposed in Fig 1a, as a function of wavelength in the range 1500 nm to 3000 nm. According to Fig 3, the transmission rate (red curve) exhibits a very high maximum, reaching 0.92 at λ = 1626 nm, indicating a resonance where the majority of the incident wave passes through the structure. This resonance is accompanied by minimal reflection (blue curve) and low absorption (yellow curve). Other resonances appear at λ=~2100 nm with T = 0.48, where reflection increases considerably, reflecting destructive interference effects. On the other hand, absorption remains low overall across the spectrum, with a slight peak at ~2096 nm, implying low losses in the structure.

### 3.2 Wave propagation characteristics of a 2D perfect periodic plasmonic structure

Fig 4a–4f displays the EFD inside the four-period defect-free MIM structure at wavelengths chosen from either the transmission bands or the bandgaps of the spectra shown in Fig 5. At 1655 nm, 2076 nm, and 2418 nm (Fig 4a, 4c and 4e), which lie in transmission bands with high transmission, low reflection, and moderate absorption, the field is strongly excited in the resonator region of each unit cell and propagates along the entire periodic chain, indicating efficient coupling of the plasmonic mode from one period to the next. The field patterns at these wavelengths show noticeable intensity in all four cells, consistent with extended Bloch-type modes that carry energy through the structure.

**Fig 4:**
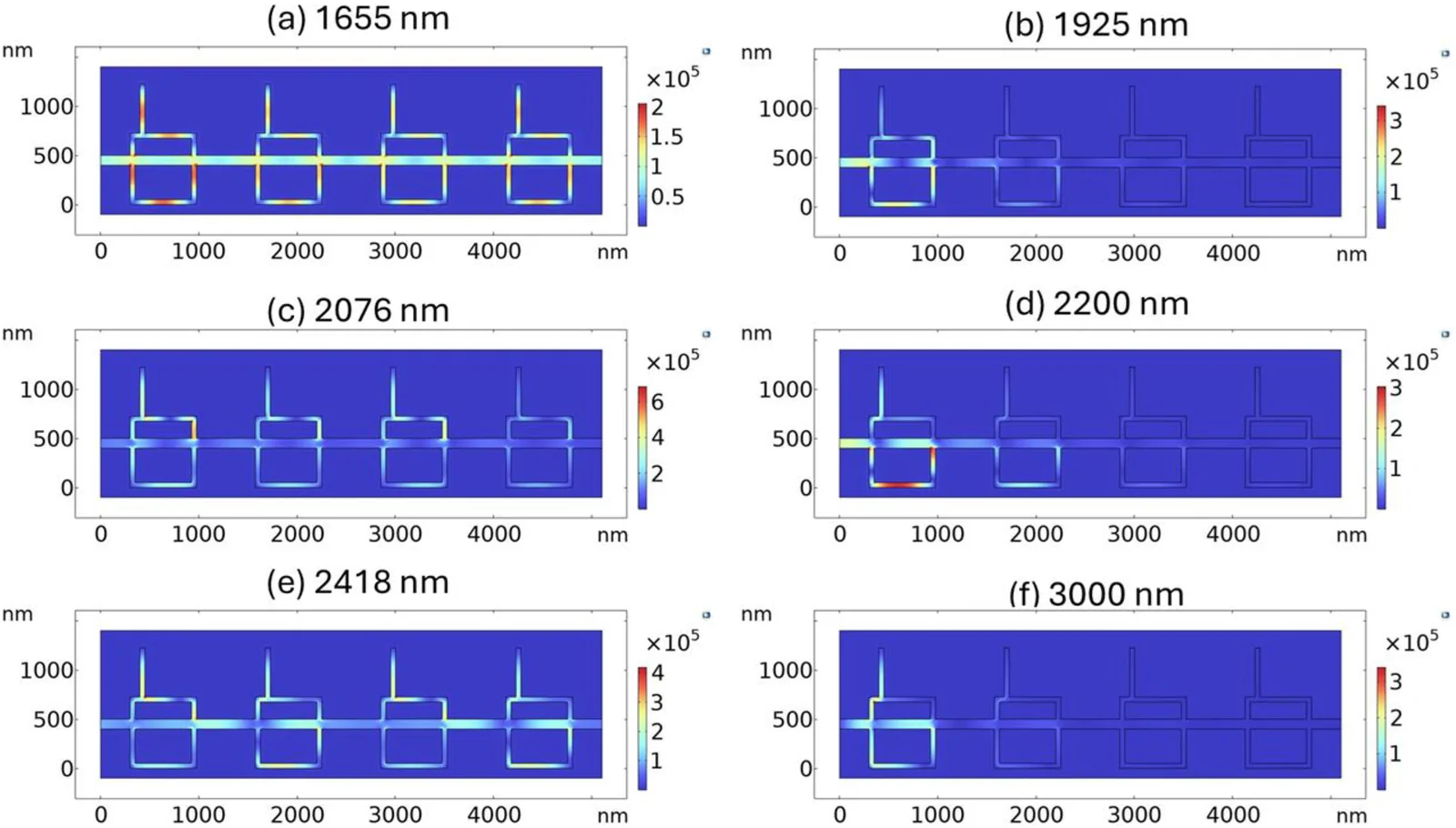
Spatial distribution of the electric field (EFD, field norm) in the periodic MIM plasmonic waveguide formed by four identical, defect-free unit cells (structure of Fig 1b) at six representative wavelengths: (a) 1655 nm, (b) 1925 nm, (c) 2076 nm, (d) 2200 nm, (e) 2418 nm, and (f) 3000 nm. The selected wavelengths correspond to transmission-band states with high T and low R at 1655 nm, 2076 nm, and 2418 nm, and to bandgap states with high R, strongly suppressed T, and low A at 1925 nm, 2200 nm, and 3000 nm, as identified from the spectra in Fig 5.

**Fig 5:**
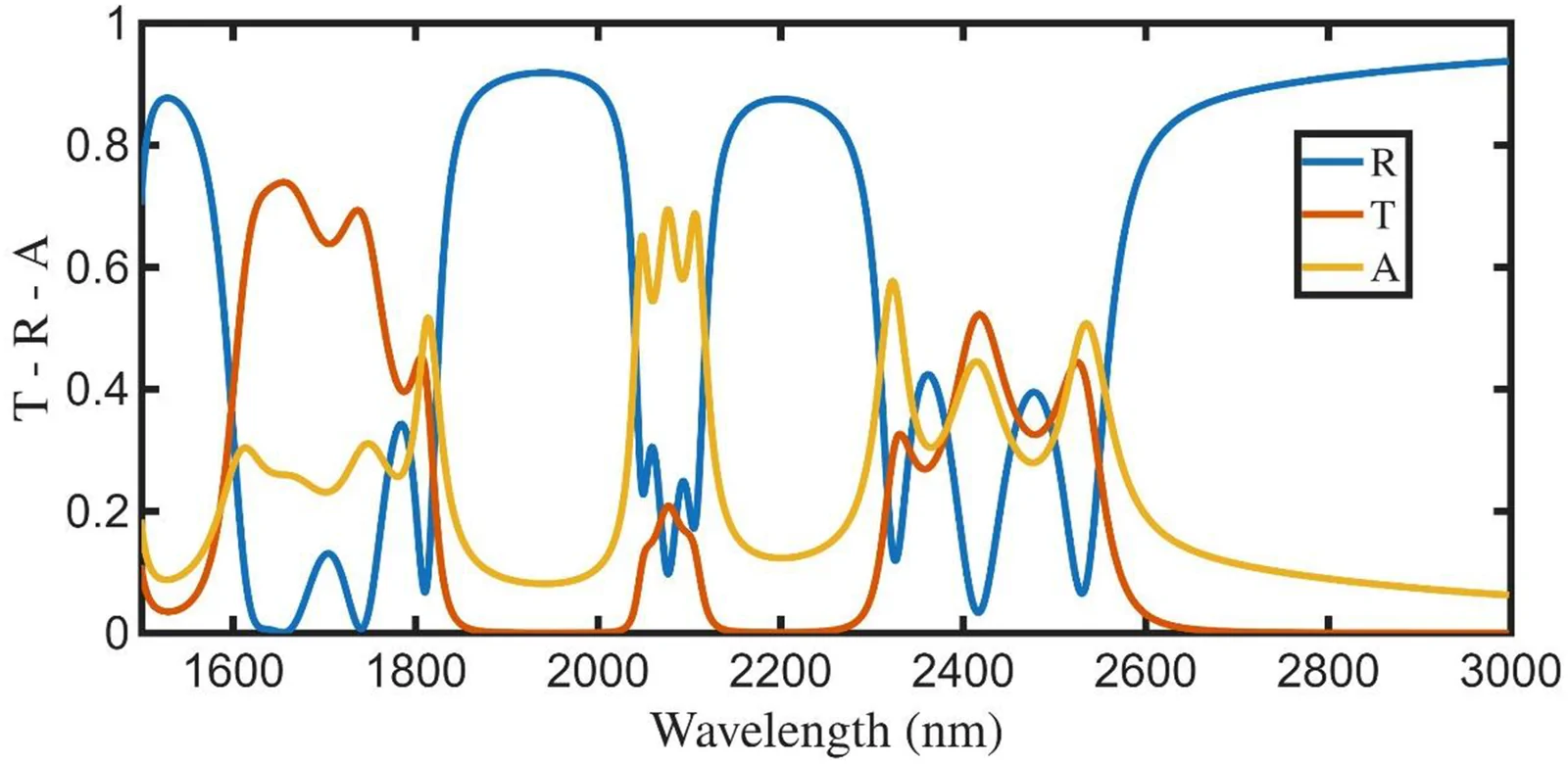
Transmission (T), reflection (R), and absorption (A) spectra of the periodic MIM plasmonic structure composed of four identical, defect-free unit cells (corresponding to Fig 1b) as a function of wavelength in the range 1500–3000 nm. The plotted curves highlight the formation of transmission bands, where T is high and R is low, and bandgaps, where R is strongly enhanced, T is strongly suppressed, and A remains comparatively small.

In contrast, at 1925 nm, 2200 nm, and 3000 nm (Fig 4b, 4d and 4f), corresponding to bandgap frequencies with maximum reflection, minimal transmission, and low absorption, the EFD is predominantly confined near the input side of the structure and decays rapidly towards the downstream unit cells. The downstream cavities are only weakly excited, evidencing strong suppression of mode propagation inside the periodic array. This spatial decay of the field is a hallmark of a photonic/plasmonic bandgap, where the periodic MIM configuration prevents the formation of propagating plasmonic modes and the incident energy is mainly reflected back rather than transmitted through the four-cell stack.

Fig 5 illustrates the transmission (T), reflection (R) and absorption (A) rates as a function of wavelength for the perfect periodic structure (Fig 1b) composed of four-unit cells. The proposed structure features three passbands separated by three width gaps in which propagation is forbidden. The first band gap appears between λ = 1863 nm and λ = 2020 nm, the second between λ = 2138 nm and λ = 2272 nm, and the third begins at λ = 2652 nm. These forbidden bands result from the increased number of unit cells. Their importance lies in their ability to strongly and locally confine defect modes. Absorption (yellow curve) remains moderate overall but reaches significant values in certain spectral ranges, suggesting the presence of dissipative metallic material (Ag) responsible for losses.

These forbidden bands arise from the Bragg-type interference of plasmonic modes in the periodic MIM–resonator lattice, which prevents certain wavelengths from propagating and leads to strong reflection [57]. The presence of well-developed band gaps is crucial for hosting sharply localized defect modes when a perturbation (defect cell) is introduced. These stop bands demonstrate that the periodic MIM–resonator structure behaves as a compact plasmonic analogue of a one-dimensional photonic crystal, while remaining fully compatible with planar MIM waveguides. Such simultaneously deep reflection bands and relatively low absorption are difficult to obtain in simple MIM waveguide–ring configurations, which typically provide only a few isolated resonances rather than extended band gaps. The presence of multiple band gaps is essential for hosting spectrally isolated defect modes in the subsequent defective structure, which is a key ingredient for achieving high sensitivity and low detection limits in sensing applications.

### 3.3 Wave propagation characteristics of a defect plasmonic structure

#### 3.3.1 Optimum defect position in periodic plasmonic structure.

Fig 6 illustrates the EFD in the four-period MIM structure when a defect cell is inserted after the second unit cell (N = 2), at the two resonant defect wavelengths corresponding to the transmission dips observed in Fig 7. At λ = 1908.9 nm (Fig 6a), the electric field is strongly confined inside and around the defect cavity, with only limited penetration into the adjacent unit cells, demonstrating the formation of a highly localized defect mode embedded in the bandgap of the periodic lattice. This intense, deep-subwavelength localization is consistent with the narrow spectral feature characterized by low transmission, high reflection, and a sharp absorption peak.

**Fig 6:**
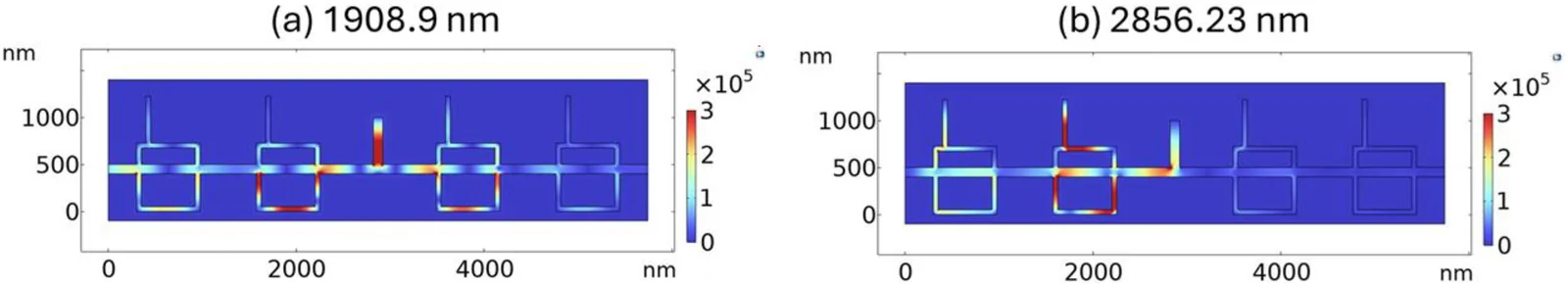
Spatial distribution of the electric field (EFD, field norm) in the periodic MIM plasmonic waveguide composed of four-unit cells with a defect cell inserted after the second period (N = 2), evaluated at the two resonant defect wavelengths corresponding to the transmission dips in Fig 7: λ = 1908.9 nm (a) and λ = 2856.23 nm (b). The color maps show the strong localization of the plasmonic field within the defect region and its coupling to the neighboring cells at these defect-mode resonances.

**Fig 7:**
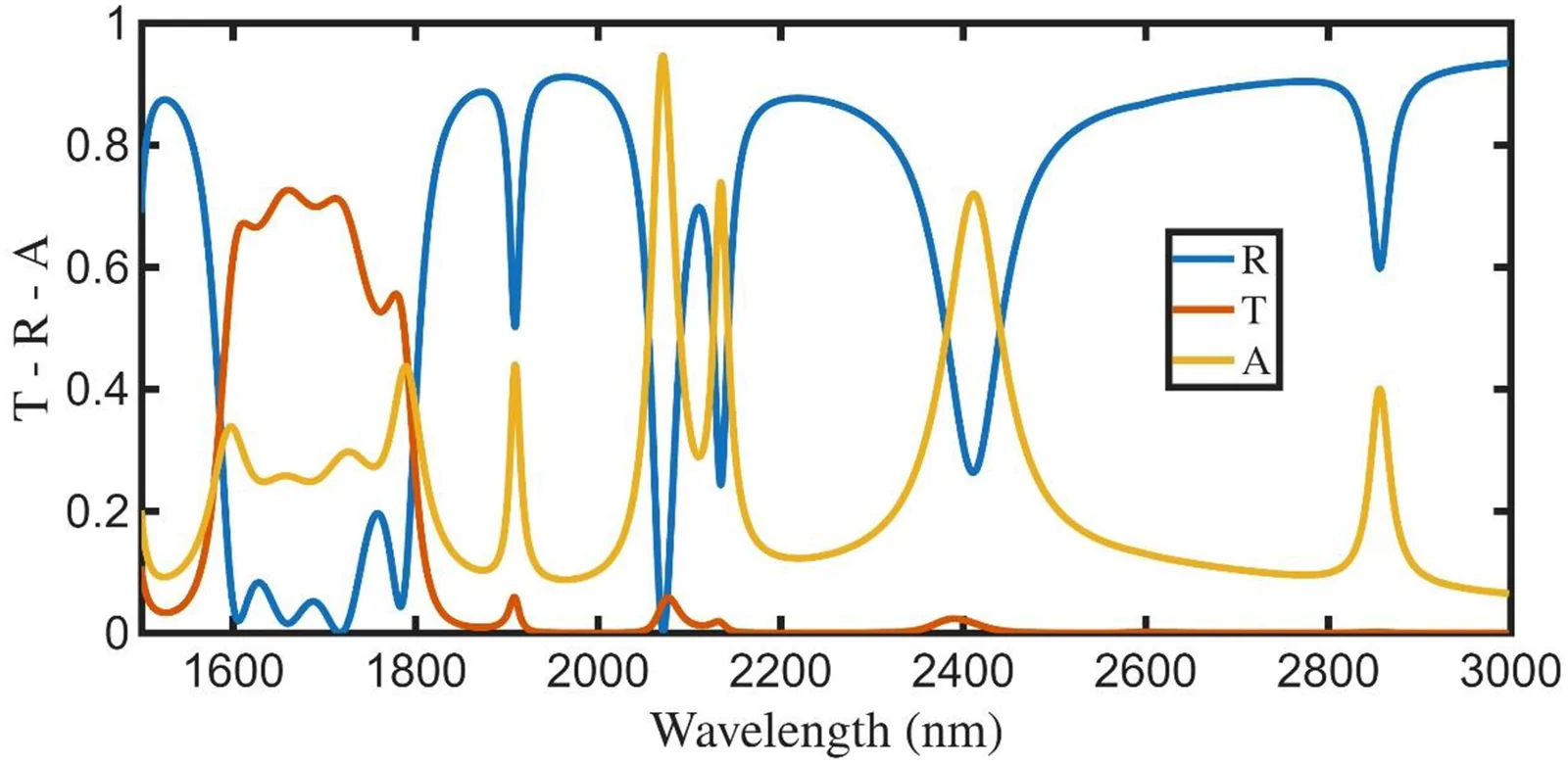
Transmission (T), reflection (R), and absorption (A) spectra of the four-period MIM plasmonic structure containing a defect cell inserted after the second unit (N = 2), plotted as a function of wavelength from 1500 nm to 3000 nm. The curves highlight two resonant defect dips in transmission at λ = 1908.9 nm and λ = 2856.23 nm, accompanied by pronounced absorption peaks and dominant reflection over most of the spectral range.

At λ = 2856.23 nm (Fig 6b), the field remains centered on the defect but exhibits a broader spatial profile, extending more visibly towards the neighboring resonators and with a lower peak amplitude than at 1908.9 nm. This pattern indicates a second defect-induced resonance that is more weakly confined and more strongly coupled to the surrounding cells, in agreement with the broader absorption response in Fig 7. Taken together, the two EFD maps confirm that the introduced defect supports multiple resonant modes at different wavelengths, each with a distinct degree of spatial localization and interaction with the finite plasmonic lattice.

Fig 7 presents the spectral response of the periodic MIM structure when a defect cell is introduced after the second period, showing how the defect profoundly modifies the T–R–A characteristics. For wavelengths above approximately 1800 nm, the transmission remains very low over broad intervals, while reflection is close to unity, indicating that the defective structure behaves globally as a highly reflective plasmonic mirror. Superimposed on this high-reflection background, two narrow resonant dips in transmission appear at λ = 1908.9 nm and λ = 2856.23 nm, which correspond to defect-induced modes supported by the modified cavity.

At these wavelengths, the absorption spectrum exhibits sharp peaks, confirming that the incident energy is efficiently coupled into localized plasmonic resonances centered at the defect cell rather than being simply reflected. The coexistence of high reflection off resonance and strong absorption at the defect wavelengths evidences the hybrid nature of the response: the periodic lattice opens bandgaps that block propagation, while the defect introduces discrete resonant states inside these gaps. These localized defect modes, associated with the deep transmission dips and absorption maxima, are precisely the resonances whose spatial field distributions are depicted in Fig 6.

From a physical perspective, the localized defect resonance observed in our periodic MIM structure shares the concept of defect modes in one-dimensional photonic crystals but differs in its origin and properties. In our case, the resonance is strongly influenced by the dual-resonator geometry embedded in a finite plasmonic lattice, leading to deep-subwavelength confinement and pronounced local field enhancement in the metal–insulator region. This behaviour contrasts with defect modes in classical photonic crystals, which are typically distributed over larger dielectric regions and exhibit weaker confinement; as a result, the present plasmonic defect mode is more sensitive to small refractive index changes, which is reflected in the reported high sensitivity and low DL values.

Fig 8 shows the wave propagation properties in a periodic structure composed of six cells, including a defective cell located at N = 3. Analysis of the transmission (T), reflection (R), and absorption (A) coefficients show that reflectance throughout the MIM structure is very high at the wavelength of λ = 1908.8 nm and λ = 2856.8 nm, for which the reflection coefficients reach R = 0.85 and R = 0.89, respectively. On the other hand, the structure is more absorbent in the wavelength ranges [2000 nm – 2150 nm] and [2300 nm – 2600 nm]. The results presented in Figs 7 and 8 were obtained with a view to determining the optimum position of the defect within the periodic structure. From the analysis of these Figs 7 and 8, we conclude that the optimum period is N = 2. From an application standpoint, choosing N = 2 as the defect position offers a practical trade-off between compact footprint and strong defect mode localization. In contrast to earlier MIM sensors where a single cavity is side-coupled to an infinite or semi-infinite waveguide, here the finite periodic environment and optimized defect position enhance the field confinement around the defect and increase the separation between neighboring resonances. This engineered plasmonic environment is directly responsible for the narrow, well-isolated defect mode that underpins the high sensing performance reported in Section 3.3.2.

**Fig 8:**
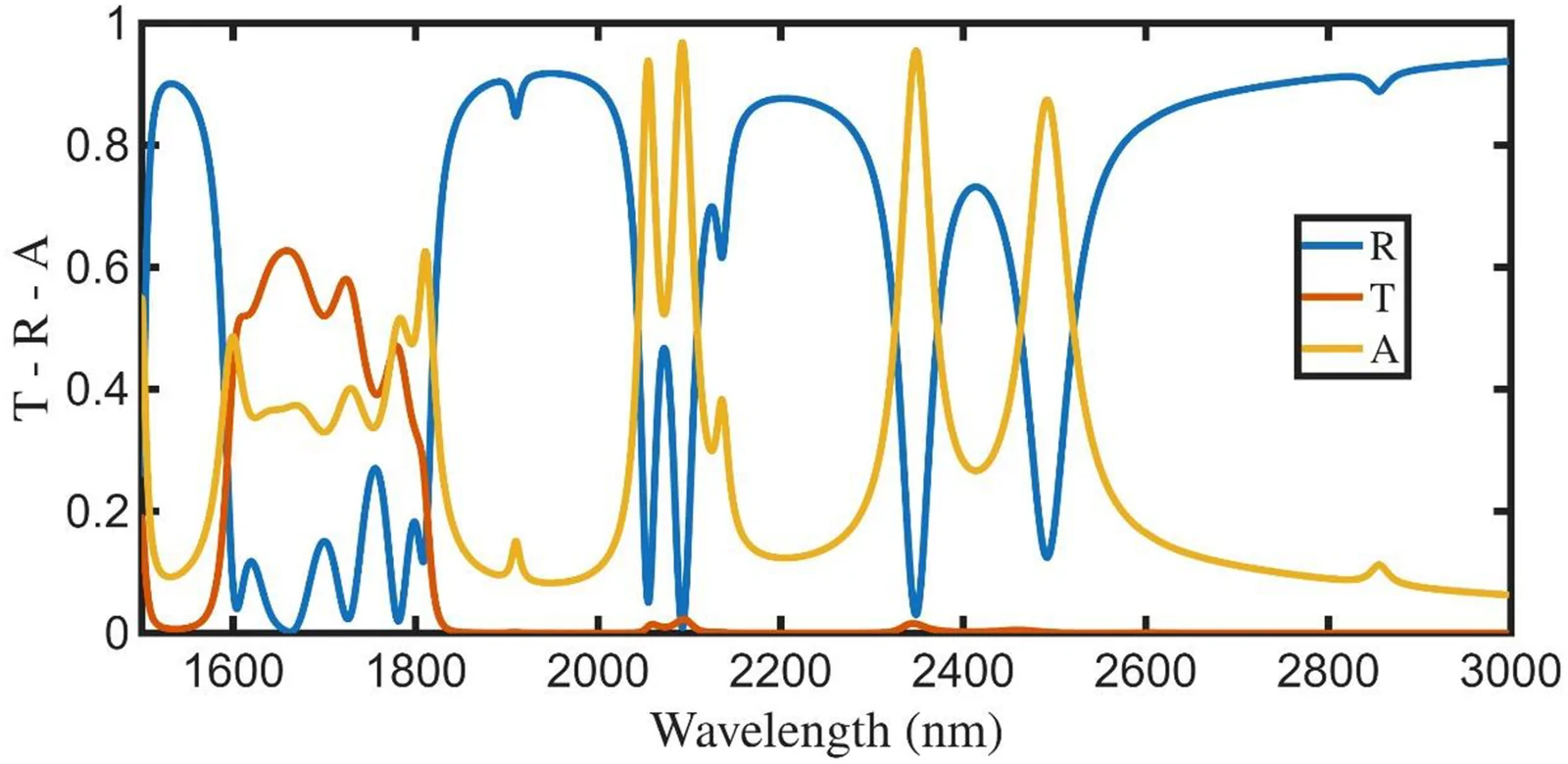
Transmission (T), reflection (R), and absorption (A) spectra of the periodic MIM plasmonic structure composed of six-unit cells with a defect cell inserted after the third period (N = 3), plotted as a function of wavelength from 1500 nm to 3000 nm.

In the following, we will consider that the defect is located between N = 2 and seek to optimize other parameters in order to design a simple, high-performance gas sensor, focusing in particular on criteria such as sensitivity, detection limit (DL), and figure of merit (FOM).

#### 3.3.2 Optimum defect resonator width and height.

In Fig 9, we investigate the influence of the defect-cavity width $w_{\text{0}}$ on the position of the defect resonance observed in reflection. Increasing $w_{\text{0}}$ modifies the lateral confinement of the SPP mode in the defect cell and changes the effective index $n_{\text{eff}}$ experienced by the mode within the MIM slot. From a resonator viewpoint, the defect acts as a finite plasmonic cavity that satisfies a phase-matching condition of the form

**Fig 9:**
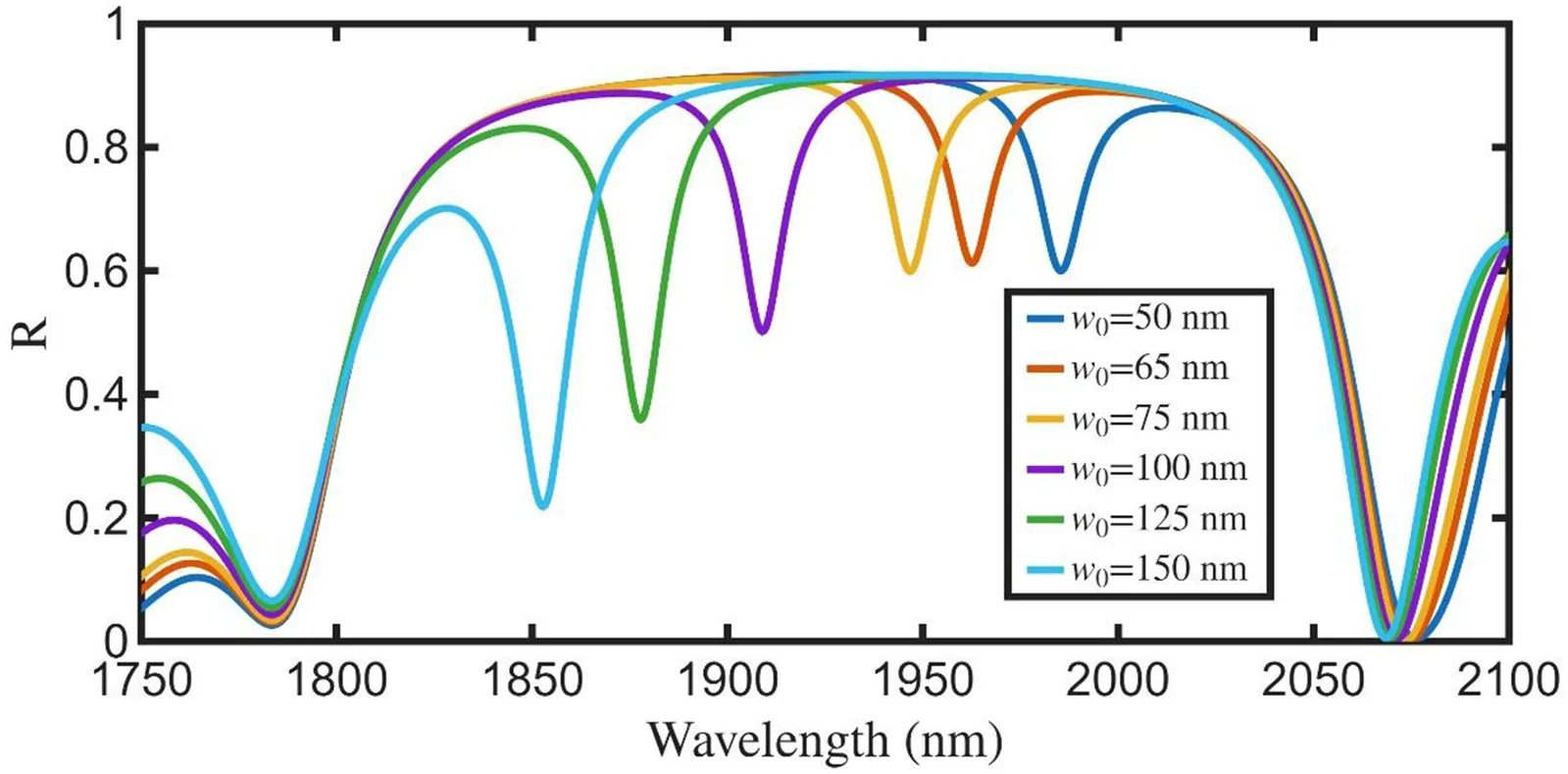
Reflectance spectra of the periodic MIM plasmonic structure with a defect cell placed at N = 2 for different widths $w_{0}$ of the defect resonator (50–150 nm), showing the shift of the defect-mode resonance as a function of $w_{0}$.

$\text{2}\;n_{\text{eff}}\;L_{\text{eff}}=m\;\lambda_{\text{res}},$ where $L_{\text{eff}}$ is the effective cavity length and m is an integer resonance order. When $w_{\text{0}}$ is varied, both $n_{\text{eff}}$ and $L_{\text{eff}}$ are slightly modified, so the phase condition is fulfilled at different $\lambda_{\text{res}}$. This explains the observed systematic shift of the defect dip in Fig 9. Importantly, even small changes in $w_{\text{0}}$ induce clearly resolvable shifts in $\lambda_{\text{res}}$, which confirms that the resonance is governed by strong plasmonic confinement in the defect cell and is highly sensitive to the effective index variation.

According to Fig 9, the defect mode shifts to shorter wavelengths as the width ${\text{w}}_{\text{0}}$ increases. Furthermore, a small variation in width results in a significant difference in wavelength between two successive defect modes, avoiding the problem of overlapping between these modes and making our system a good candidate for detection and filtering. Thus, a width of ${\text{w}}_{\text{0}}$ = 100 nm was identified as the optimum value, as it offers a good compromise between clear spectral localization of the defect mode and sufficient separation between successive modes. ${\text{w}}_{\text{0}}$ of 100 nm will be retained for following studies. This strong and monotonic tuning of the defect mode with w₀, combined with the absence of overlapping resonances, is particularly advantageous compared to many previously reported MIM filters, where closely spaced modes complicate the unambiguous tracking of resonance shifts for sensing purposes.

Fig 10 presents the reflectance spectra of the periodic structure with a single defect cell for different heights $h_{\text{0}}$ of the defect resonator, with the defect position fixed at N=2 and the defect width at $w_{\text{0}}=\text{1}\text{0}\text{0}$ nm. A similar mechanism is responsible for the resonance shifts observed when the defect height $h_{\text{0}}$ is varied (Fig 10). Changing $h_{\text{0}}$ modifies the vertical dimension of the MIM cavity and thus the overlap of the plasmonic mode with the metal and dielectric regions. This changes the effective index $n_{\text{eff}}$ of the guided SPP and the effective cavity length $L_{\text{eff}}$ along the propagation direction in the defect region. As a consequence, the phase accumulation condition for resonance is satisfied at different wavelengths, which manifests as a systematic shift of the defect dip when $h_{\text{0}}$ increases or decreases. In other words, the resonance tuning with $h_{\text{0}}$ is a direct consequence of the sensitivity of the plasmonic confinement to the vertical geometry of the defect cavity.

**Fig 10:**
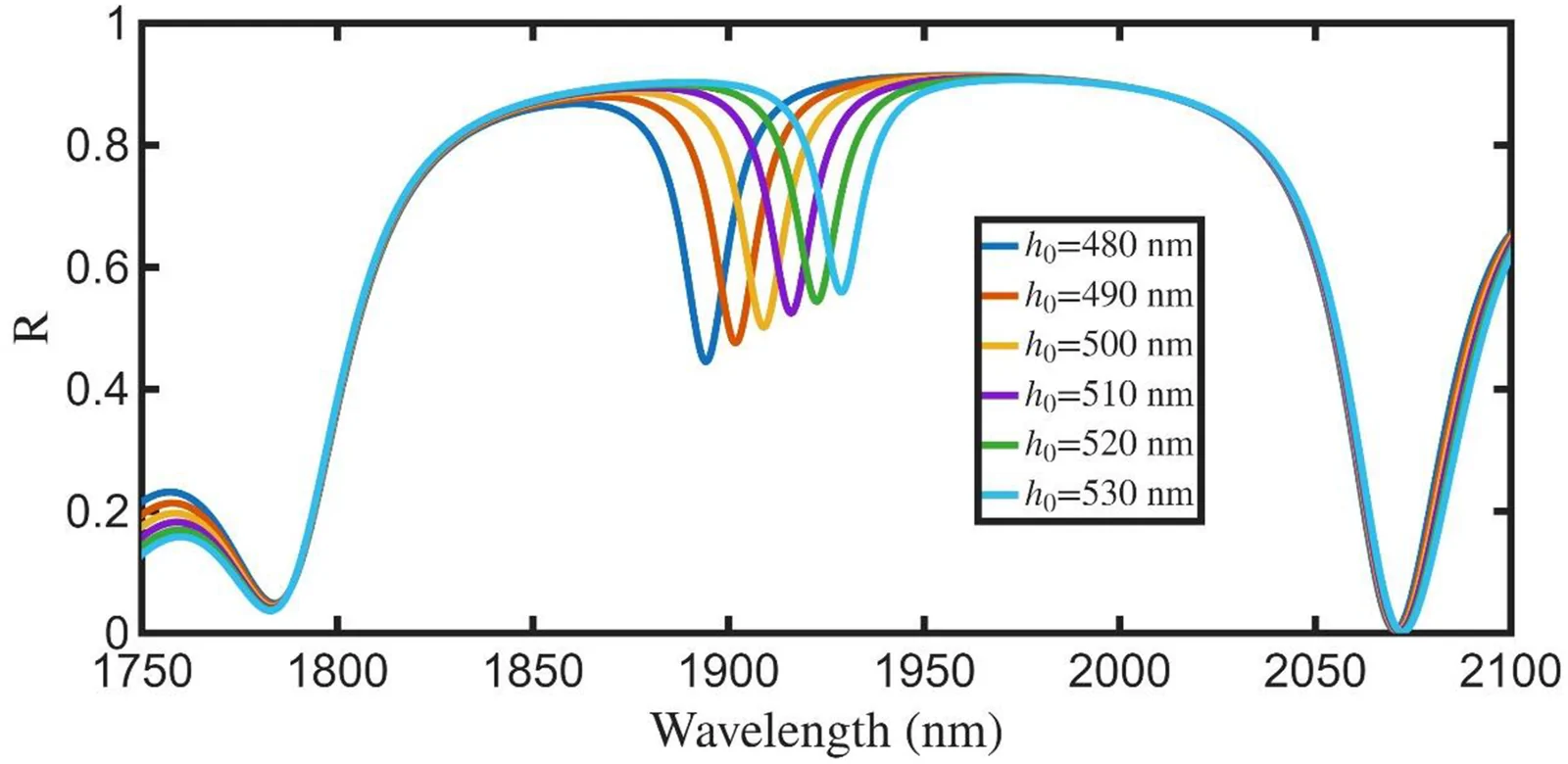
Reflectance spectra of the periodic MIM structure with a defect cell at position N=2 for different heights $h_{0}$ of the defect resonator, with the defect width fixed at $w_{0}=100$ nm.

As $h_{\text{0}}$ increases from 480 nm to 530 nm, this defect-induced dip shifts monotonically towards longer wavelengths and its depth changes, showing that the optical path length in the defect cavity is being tuned by the height of the resonator. Among the curves, the case $h_{\text{0}}=\text{5}\text{3}\text{0}$ nm produces a dip that is located closest to the center of the band gap. Locating the resonance near the band-gap center increases the field confinement in the defect and typically maximizes the sensitivity of the resonance wavelength to changes in the refractive index of the gas in the cavity [19,58–60]. For this reason, $h_{\text{0}}=\text{5}\text{3}\text{0}$ nm is selected as the optimal height and is used in the subsequent sensing analysis, confirming that the defect resonator height is an effective tuning parameter rather than a passive geometric detail.

#### 3.3.3 Essential characteristics of the infrared gas sensor.

The following equations demonstrate how to calculate key performance metrics, including sensitivity (S), figure of merit (FoM), quality factor (Q), and detection limit (DL) [61,62]:


$$\begin{array}{c} S=\frac{\Delta \lambda_{R}}{\Delta n_{gas}}, \end{array}$$ (6)

$$\begin{array}{c} FoM=\frac{S}{FWHM}, \end{array}$$ (7)

$$\begin{array}{c} \begin{array}{c} Q=\frac{\lambda_{R}}{FWHM} \end{array}, \end{array}$$ (8)

$$\begin{array}{c} LoD=\frac{\lambda_{R}}{\text{2}\text{0}\;S\;Q}. \end{array}$$ (9)

where the FWHM is the bandwidth for air sample.

In Fig 11a, we examine how the reflectance spectrum evolves when the refractive index n of the gas in the defect resonator increases from 1.00 to 1.10. Each curve exhibits a single, narrow reflectance dip associated with the defect mode, and this dip shifts systematically towards longer wavelengths as n increases. This red-shift arises because a higher refractive index increases the effective optical path length in the defect cavity, so a larger wavelength is required to satisfy the resonance condition. The shift between successive curves for a step Δn=0.02 RIU is clearly larger than the linewidth of each dip, so the corresponding minima remain well separated and do not overlap. This pronounced, monotonic displacement of the resonance wavelength with n means that even small refractive index changes can be resolved accurately, which is essential for high-precision gas sensing.

**Fig 11:**
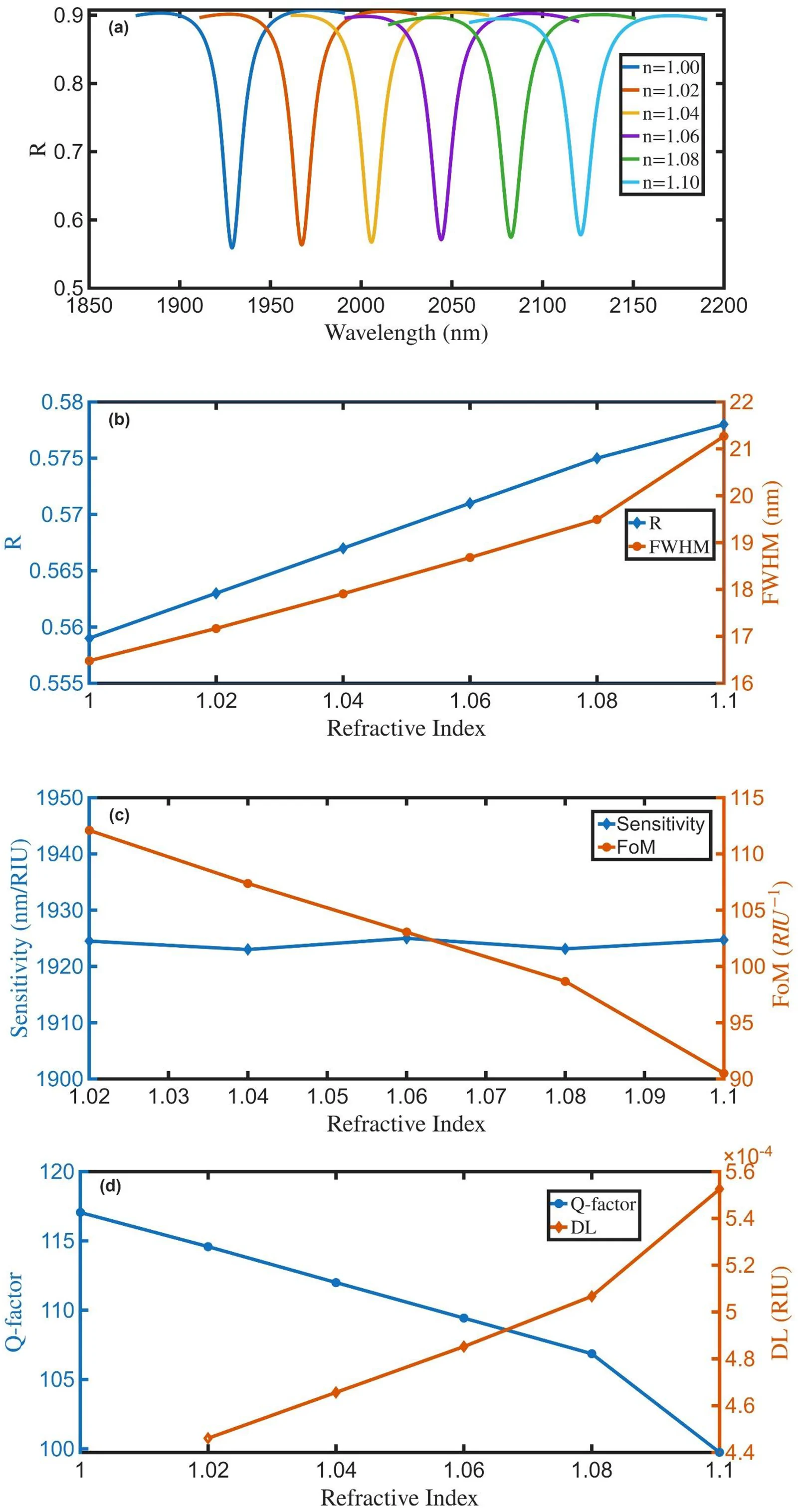
(a) The reflectance, (b), (c), and (d) of the proposed structure with defect cell at different refractive indices of gas sample at N=2, ${𝐰}_{1}$=100 nm, ${𝐰}_{2}$=50 nm, ${𝐰}_{3}$=50 nm, ${𝐰}_{0}$=100 nm, ${𝐝}_{1}$=1275 nm, ${𝐝}_{2}$=625 nm, ${𝐡}_{1}$=500 nm, ${𝐡}_{2}$=725 nm, ${𝐡}_{3}$=625 nm, ${𝐡}_{4}$=1500 nm, ${𝐡}_{0}$= 530 nm, and ${𝐝}_{0}$=${𝐝}_{1}/2$ Fig 11d shows how the Q-factor and detection limit (DL) vary with the refractive index n. The Q-factor remains high over the whole range but decreases approximately linearly from about 117 at n=1.00 to about 100 at n=1.10, indicating a gradual reduction of resonance sharpness as the index increases. At the same time, the DL exhibits the opposite trend, rising from roughly $\text{4}.\text{4}\times {\text{1}\text{0}}^{-\text{4}}$ RIU to about $\text{5}.\text{5}\times {\text{1}\text{0}}^{-\text{4}}$ RIU, which reflects the combined effect of slightly broader resonances and the FoM decrease observed in Fig 11c. These results confirm that the sensor operates in a regime where Q remains comfortably above 100 for all tested refractive indices, but the best detection performance is obtained near the lower-index side (around n≈1.00–1.02), where Q is highest and DL is minimal.

Fig 11b summarizes how the reflectance at the defect resonance and the corresponding FWHM evolve with the refractive index n. Both quantities increase almost linearly as n varies from 1.00 to 1.10: the reflectance rises from about 0.559 to 0.578, while the FWHM broadens from roughly 16.5 nm to 21.3 nm. This trend shows that higher refractive indices lead to a slightly stronger background reflection and a noticeably wider resonance dip, which reduces the spectral sharpness of the defect mode. The gradual broadening of the FWHM is consistent with a moderate reduction in the Q-factor and, consequently, a small loss in spectral resolution at larger n, in agreement with the behaviour of Q and DL reported in Fig 11d.

Fig 11c shows the dependence of the sensitivity S and the figure of merit (FoM) on the refractive index n. The sensitivity remains high and nearly constant over the interval n=1.02–1.10, varying only slightly from about 1923–1925 nm/RIU, which indicates that the resonance wavelength shift per refractive index unit is essentially preserved across this range. In contrast, the FoM decreases approximately linearly as n increases, dropping from about 112 ${\text{RIU}}^{-\text{1}}$ at n=1.02 to roughly 91 ${\text{RIU}}^{-\text{1}}$ at n=1.10, reflecting the combined effect of a modestly broadened resonance (larger FWHM) and the nearly constant sensitivity. This behaviour means that, while the sensor continues to provide a strong wavelength response to refractive index changes, its spectral selectivity and overall performance are slightly better in the lower-index region near n≈1.02.

The combination of a high sensitivity on the order of 1920 nm/RIU, a Q-factor above 100, and a minimum detection limit of $\text{2}.\text{5}\times {\text{1}\text{0}}^{-\text{4}}$ RIU, obtained near n≈1.02, shows that the defect mode supported by the proposed dual-resonator periodic structure is both sharp and highly responsive to refractive index variations. Achieving this simultaneous performance in S, FoM, Q and DL is challenging for single-ring MIM sensors and for conventional photonic crystal structures, where improving one metric often degrades another.

Table 2 presents a comparative study of the sensitivity and detection limit reported for several previously published plasmonic and MIM-based sensors together with the values obtained for the present design. From this table, it is evident that our device offers both a higher sensitivity (about 1900–2000 nm/RIU in the operating range) and a lower detection limit (down to $\text{2}.\text{5}\times {\text{1}\text{0}}^{-\text{4}}$ RIU) than many of the referenced structures. These results indicate that the proposed metasurface plasmonic ring-resonator configuration is a strong candidate for infrared gas sensing and filtering applications.

As summarized in Table 2, the proposed device exhibits high overall performance compared with many previously reported plasmonic MIM and photonic crystal sensors. In several earlier MIM-based designs, the sensitivity typically lies between a few hundred and about 1600 nm/RIU, and the detection limit is often on the order of ${\text{1}\text{0}}^{-\text{3}}$–${\text{1}\text{0}}^{-\text{2}}$ RIU, whereas our metasurface ring-resonator structure reaches sensitivities around 1920 nm/RIU in the operating range and a minimum detection limit of $\text{2}.\text{5}\times {\text{1}\text{0}}^{-\text{4}}$ RIU. This improvement can be attributed to the combined effect of periodic band-gap engineering, dual-resonator coupling and careful defect optimization, which together produce a sharp and tunable defect mode in the infrared region.

Although the present study relies on numerical FEM simulations, the proposed MIM plasmonic structure is compatible with established nanofabrication techniques. Similar MIM waveguides and ring or loop resonators have been realized experimentally using electron-beam lithography, thin-film metal deposition and lift-off or dry etching on planar substrates [71]. The feature sizes required in our design (down to several tens of nanometers) are within reach of these processes, indicating that the periodic dual-resonator architecture could be implemented in practice. As a next step, we plan to fabricate and characterize the sensor experimentally and to analyze in detail how fabrication tolerances and metal losses affect the sensitivity and detection limit.

## 4. Conclusion

This study demonstrates the significant potential of resonant photonic-plasmonic structures for high-performance filtering and sensing applications. Through detailed finite element method (FEM) simulations, we have shown that precise optimization of optical and geometric parameters, particularly in a looped parallel waveguide configuration with an integrated resonator, can drastically enhance device efficiency. Our findings reveal that tailored adjustments to structural dimensions critically influence transmission spectra and resonance behavior, enabling fine-tuned control over light-matter interactions. Notably, the proposed plasmonic sensor exhibits remarkable sensitivity, detecting refractive index changes as minute as 0.02 RIU with an exceptional peak sensitivity of 1920 nm/RIU. These results underscore the viability of this design for advanced photonic sensing systems, offering promising applications in biomedical diagnostics, environmental monitoring, and integrated optoelectronic circuits. Future work could explore experimental validation and scalability for practical implementation.
